# Cell-type-specific interrogation of CeA *Drd2* neurons to identify targets for pharmacological modulation of fear extinction

**DOI:** 10.1038/s41398-018-0190-y

**Published:** 2018-08-22

**Authors:** Kenneth M. McCullough, Nikolaos P. Daskalakis, Georgette Gafford, Filomene G. Morrison, Kerry J. Ressler

**Affiliations:** 1 Division of Depression and Anxiety Disorders, McLean Hospital, Department of Psychiatry, Harvard Medical School, Boston, MA USA; 2 Department of Psychiatry, and Behavioral Sciences, Behavioral Neuroscience, Emory University, Atlanta, GA USA; 3 VA Boston Healthcare System, Boston, MA USA; 4 Behavioral Science Division, National Center for PTSD, Boston, MA USA; 5 Department of Psychiatry, Boston University School of Medicine, Boston, MA USA

## Abstract

Behavioral and molecular characterization of cell-type-specific populations governing fear learning and behavior is a promising avenue for the rational identification of potential therapeutics for fear-related disorders. Examining cell-type-specific changes in neuronal translation following fear learning allows for targeted pharmacological intervention during fear extinction learning, mirroring possible treatment strategies in humans. Here we identify the central amygdala (CeA) *Drd2*-expressing population as a novel fear-supporting neuronal population that is molecularly distinct from other, previously identified, fear-supporting CeA populations. Sequencing of actively translating transcripts of *Drd2* neurons using translating ribosome affinity purification (TRAP) technology identifies mRNAs that are differentially regulated following fear learning. Differentially expressed transcripts with potentially targetable gene products include *Npy5r, Rxrg, Adora2a, Sst5r, Fgf3, Erbb4, Fkbp14, Dlk1*, and *Ssh3*. Direct pharmacological manipulation of NPY5R, RXR, and ADORA2A confirms the importance of this cell population and these cell-type-specific receptors in fear behavior. Furthermore, these findings validate the use of functionally identified specific cell populations to predict novel pharmacological targets for the modulation of emotional learning.

## Introduction

The amygdala is a mediator of the acquisition and expression of learned associative fear^1,2^. Composed primarily of GABAergic medium spiny neurons, the central amygdala (CeA) is intimately involved in controlling the expression of fear-related behaviors^3,4^. Each of the CeA’s three main sub-nuclei (lateral capsular (CeC), lateral (CeL), and medial (CeM)) play distinct roles in specific behaviors and contain molecularly distinct sub-populations that have further behavioral specializations^5–10^. In the present set of experiments, we utilized Pavlovian fear conditioning, a paradigm used extensively for studying associative fear memories formed by the pairings of conditioned stimuli (CS; e.g. a tone) and unconditioned stimuli (US; e.g. a mild foot shock)^11–13^. Learned fearful associations may be ‘extinguished’ with additional unreinforced presentations of the CS alone, a process that closely resembles the clinical practice of exposure therapy used in treating individuals with posttraumatic stress disorder (PTSD). A promising area of treatment in PTSD includes the pharmacological enhancement of exposure-based therapies^14^. The aim of this study was to harness cell-type-specific molecular techniques in order to identify more specific and effective pharmacotherapies for the treatment of fear-related disorders.

Foundational research as well as more recent analyses highlight the striatum-like nature of the central amygdala^15^. Striatal dopamine receptor 1 (*Drd1*) populations (direct pathway neurons) promote movement, while dopamine receptor 2 (*Drd2*) populations (indirect pathway neurons) inhibit movement^16,17^. Within the posterior CeA, it has been reported that corticotropin releasing factor (*Crh*), tachykinin 2 (*Tac2*), somatostatin (*Sst*), and neurotensin (*Nts*) expressing populations are contained within the larger *Drd1* expressing neuron population that promotes directed motivational behaviors under certain conditions^18,19^. Conversely, within the anterior CeC, the protein kinase C-δ (*Prkcd*) and calcitonin receptor-like (*Calcrl*) co-expressing population has been reported to be a sub-population of *Drd2* neurons mediating defensive behaviors or inhibiting motivated behaviors^19,20^. Given its potential role in fear behavior, the CeA *Drd2* expressing population is a high value target for translational investigation.

The dopaminergic system is well known for its role in appetitive learning; however, more recently it has been recognized for its importance in fear acquisition and fear extinction learning^21–23^. Perturbations in the dopaminergic system have been implicated in the disease etiologies of several human pathologies ranging from Parkinson’s disease to schizophrenia, depression and PTSD^24–26^. Although the dopamine receptor 2 (D2R) is clearly involved in fear acquisition and fear extinction learning, the literature to date has been equivocal on the role of D2R in the CeA, as different study designs demonstrate D2R antagonist administration may lead to conflicting effects^27–29^. In the present study, we separated the role of CeA *Drd2*-expressing neurons in fear behavior from that of receptor activity of D2R itself, and in doing so, identify a large number of alternative gene targets that are modulated by fear learning.

The present study takes the most translationally direct approach by behaviorally and molecularly characterizing the CeA *Drd2* neuronal population, examining translational changes in this population following a fear learning event, and then pharmacologically manipulating identified targets at a clinically relevant time point, during fear extinction. Molecular characterization of the Drd2 population clearly identifies it as a unique population that is largely non-overlapping with other, previously described CeA populations. Direct chemogenetic enhancement of excitability in CeA *Drd2* neurons resulted in significantly enhanced fear expression. Translating ribosome affinity purification (TRAP) and sequencing of actively translated RNAs in the *Drd2* neuron population following fear conditioning yielded a diverse set of genes that were differentially regulated by behavior^30,31^. These differentially regulated genes included *Adora2a*, *Rxrg, Sst5r, Npy5r, Fgf3, Erbb4, Gpr6, Fkbp14, Dlk1*, and *Ssh3*. Using the Druggable Genome database, genes with known pharmacological interaction partners were chosen and pharmacologically manipulated at a clinically relevant time point to oppose fear conditioning dependent changes, during fear extinction. Consistent with the identification of the *Drd2* expressing population as a fear expression supporting population, blockade of A_2A_R (G_αs_) or NPY5R (G_αi_) during fear extinction suppressed and enhanced fear expression, respectively. Additionally, activation of RXR enhanced fear extinction consolidation. Together these data provide promising new targets for understanding and manipulating fear processes, and also demonstrate the power of identifying novel pharmacological targets through the use of cell-type-specific approaches to amygdala circuit function.

## Results

### *Drd2* defines a distinct CeA population

**Fig. 1:**
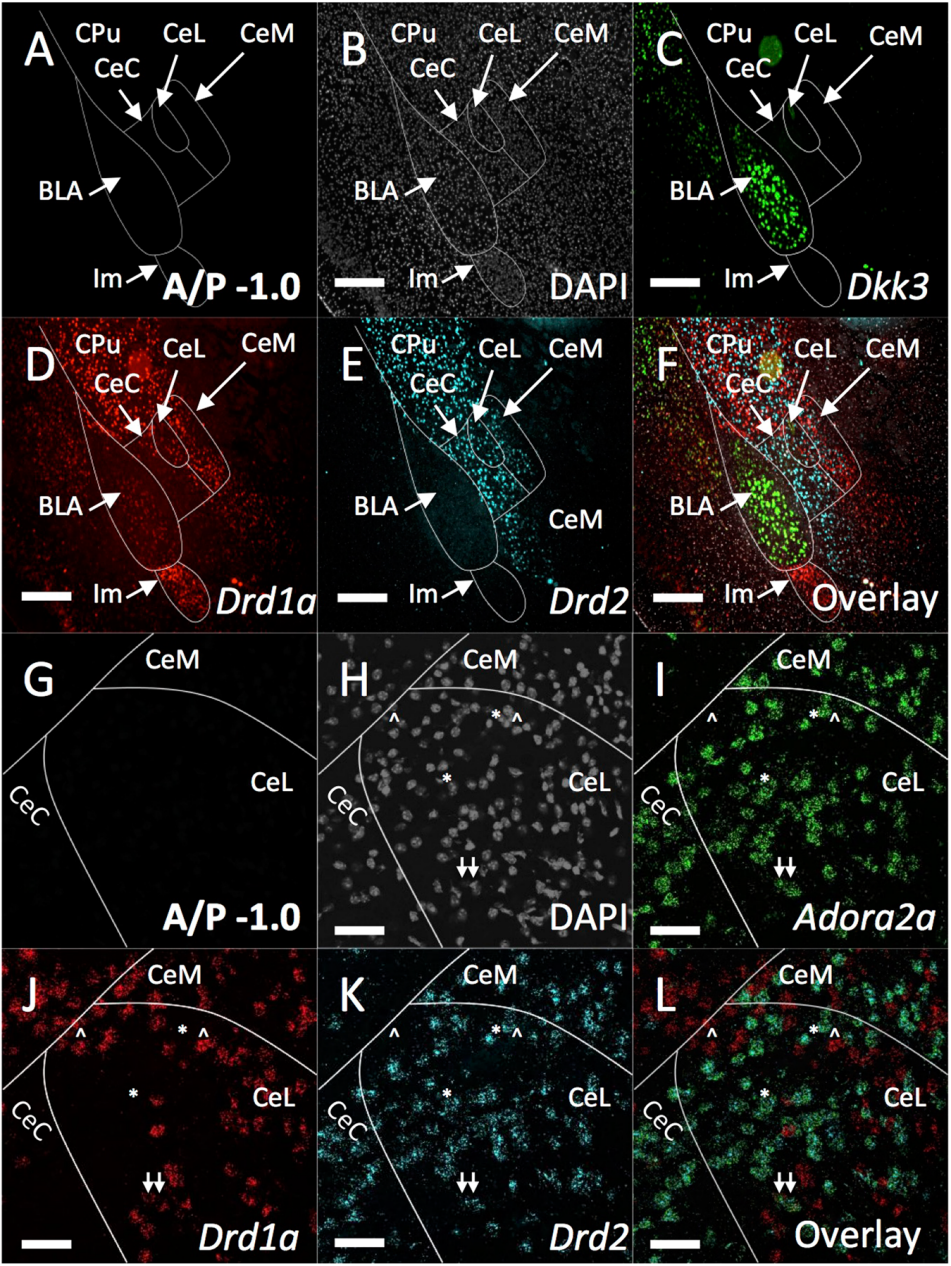
Comparison of CeA *Drd2, Drd1a*, and *Adora2a* populations. Expression of *Dkk3, Drd2, Drd1a*, and *Adora2a* were examined with FISH (RNA Scope, ACD Biosystems). **a** Schematic of amygdala compartments within the temporal lobe. **b** DAPI (grey). **c** *Dkk3* (Green) is expressed in a population of BLA pyramidal neurons. **d** *Drd1a* (Red) is expressed in striatum, weakly in some BLA cells, ITC’s (especially Im), strongly in CeL and CeM, but weakly in the CeC. **e** *Drd2* (Cyan) is expressed in striatum, CeC, CeL, but weakly in the CeM and not ITCs or BLA. **f** Overlay of **(b**–**e)**. The dorsal CeA especially CeL expresses both *Drd2* and *Drd1a* populations; however, these populations segregate primarily to the CeC and CeM, respectively, more ventrally **g**. Schematic of higher magnification anterior dorsal region of CeA. **h** DAPI (Gray). **i** *Adora2a* (Green) is expressed strongly CeC, CeL and dorsal CeM. **j** *Drd1a* (Red) is expressed strongly in CeL and CeM, but little expression is found in CeC. **k** *Drd2* (Cyan) is expressed strongly in CeC, CeL and dorsal CeM. **l** Overlay of (**h**–**k)**. *Adora2a* and *Drd2* entirely co-express. Very few examples of co-expression between *Drd1a* and either *Drd2* or *Adora2a* are found. Examples of *Drd1* expressing cells are indicated by ^. Examples of *Drd2*/*Adora2a* co-expressing cells are indicated by *. Examples of rare triple labeled *Drd1a/Drd2/Adora2a* cells are indicated by arrow. BLA basolateral amygdala, CeC central capsular amygdala, CeL central lateral amygdala, CeM central medial amygdala, CPu caudate putamen (striatum), Main Intercalated Island: Im. Staining was examined across *n* = 5 adult male animals. Scale Bar: A-F 500 μm, G-L 50 μM

Many molecularly distinct subpopulations have been identified across the CeA. Using RNAScope technology, we performed fluorescence in situ hybridization (FISH) in order to examine the *Drd2* population in relation dikkopf-related protein 3 (*Dkk3*), dopamine receptor 1a (*Drd1a*), adenosine A2A receptor (*Adora2a*), corticotropin releasing factor (*Crh*), neurotensin (*Nts*), protein kinase C-δ (*Prkcd*), somatostatin (*Sst*), and tachykinin 2 (*Tac2*). For all in situ analyses anatomical boundaries were identified by examining DAPI staining in comparison to atlases provided by the Allen Institute and Paxinos et al.^32,33^. Within the striatum (CPu), *Drd1a* and *Drd2* have expected intermingled, non-overlapping, expression patterns (Fig. 1a–f). *Dkk3* strongly labels a population of BLA primary neurons^34,35^. *Drd1a* strongly labels intercalated cell masses (ITC) especially the main intercalated island (Im), and weakly labels some BLA cells. *Drd2* does not label any BLA or ITC cells. Within the CeA at anterior positions, *Drd2* primarily labels populations in the CeC and CeL with lower expression within the CeM, while *Drd1* primarily labels populations in the CeL and CeM with less expression within the CeC^2,36,37^. At higher magnification it is clear that within the CeA *Drd1a* and *Drd2* maintain their non-overlapping expression with very few identifiable co-expressing cells (Fig. 1g–l). *Drd2* is known to strongly co-express with *Adora2a* in the striatum. Similarly, we find an almost complete co-expression of *Drd2* and *Adora2a* within central amygdala neurons (Supplemental Fig. 1).

**Fig. 2:**
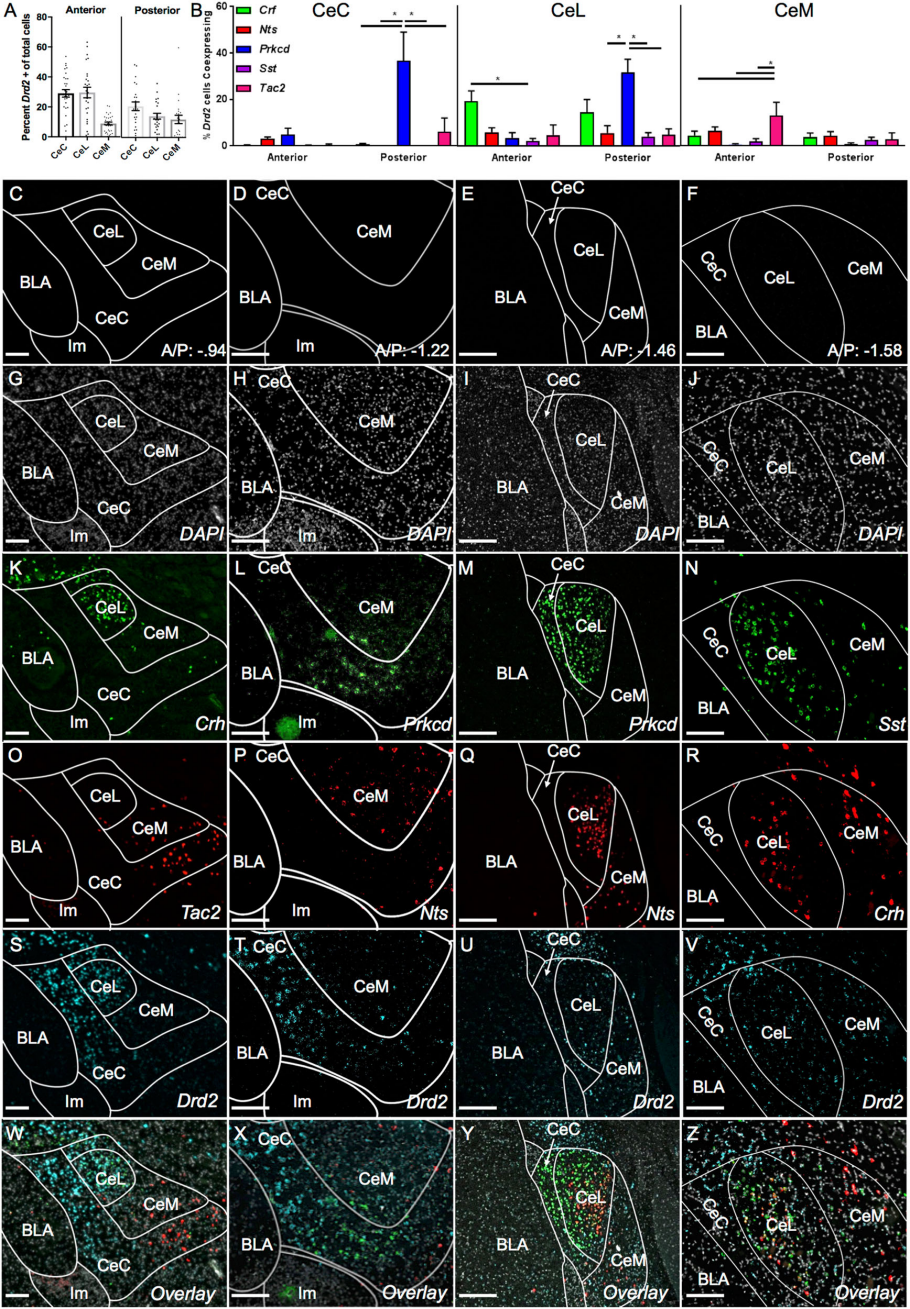
Co-localization of Drd2 with *Crh, Nts, Prkcd, Sst*, and *Tac2*. *Drd2* does not strongly co-express with any markers examined in anterior CeA. *Drd2* moderately co-expresses with *Prkcd* in posterior CeC and CeL. **a** Density of *Drd2* cell population across anterior and posterior CeA represented as a percentage of total DAPI-labeled cells. The strongest *Drd2* expression is found in anterior CeC and CeL. **b** Quantification of *Drd2* co-expression with different CeA markers at anterior and posterior positions as a percentage of total *Drd2* expressing cells (CeC: two-way ANOVA with anterior vs. posterior set as row factor (F(1,79) = 13.2), *p* = .0005) and individual RNAs set as column factor (F(4,79) = 16.19, *p* < .0001). Interaction (F(4,79) = 10.56, *p* < .0001) and Sidak’s multiple comparisons test within row: posterior: Crh vs. Prkcd *p* < .0001, Nts vs. Prkcd *p* < .0001, Sst vs. Prkcd *p* < .0001, and Tac2 vs. Prkcd *p* < .0001; CeL two-way ANOVA with anterior vs. posterior set as row factor (F(1,74) = 2.817), *p* = .0975) and individual RNAs set as column factor (F(4,74) = 5.288, *p* < .0008). Interaction (F(4,74) = 3.901, *p* < .0063) and Sidak’s multiple comparisons test within row: anterior: Crh vs. Sst *p* < .05, posterior: Nts vs. Prkcd *p* < .005, Sst vs. Prkcd *p* < .0005, Tac2 vs. Prkcd *p* < .05; CeM: two-way ANOVA with anterior vs. posterior set as row factor (F(1,77) = 3.024), *p* = .086) and individual RNAs set as column factor (F(4,77) = 2.578, *p* < .05). Interaction (F(4,77) = 1.456, *p* = .22) and Sidak’s multiple comparisons test within row: Posterior: Crh vs. Tac2 *p* < .04, Tac2 vs. Prkcd *p* < .005, Tac2 vs Sst *p* < .01). **c**–**f** Map of CeA at A/P: −0.94, −1.22, −1.46, and −1.58, respectively. **g**–**j** DAPI (Gray) expression in at A/P: −0.94, −1.22, −1.46, and −1.58, respectively. **k** *Crh* (Green) is found primarily in CeL at A/P: −.84. **l** *Prkcd* (Green) is found in a isolated population at the ventral aspect of the CeC at A/P −1.22. **m** *Prkcd* (Green) is densely expressed in CeC and CeL at A/P: −1.46. **n** *Sst* (Green) is densely expressed in CeL and more diffusely in CeM at A/P: −1.58. **o** (Red) *Tac2* is expressed in ventral CeC and CeM at A/P: −0.94. **p** *Nts* (Red) is expressed almost exclusively in CeM at A/P: −1.22. **q** *Nts* (Red) is expressed densely in CeL and diffusely in CeM at A/P: −1.46. **r** *Crh* (Red) is expressed densely in CeL and more diffusely in CeM at A/P: −1.58. **s** *Drd2* (Cyan) is expressed strongly in CeC and CeL and more weakly in CeM at A/P: −.82. **t** *Drd2* (Cyan) is expressed strongly in CeC and CeL and more weakly in CeM at A/P: −0.94. **u**, **v** *Drd2* (Cyan) is expressed more diffusely throughout CeA at A/P −1.46 and −1.58, respectively. **w**–**z** Overlay of **g**–**v**. BLA basolateral amygdala, CeC central capsular amygdala, CeL central lateral amygdala, CeM central medial amygdala, Main Intercalated Island: Im. Staining was quantified across *n* = 5 male mice with a minimum of *n* = 8 amygdala examined. Scale bar: **a**–**z** 100 μm

The anterior to posterior (A/P) position within the CeA has emerged as a strong potential variable when examining the behavioral functions of CeA neurons^19^. Therefore, the distribution of *Drd2, Drd1a*, and *Adora2a* expressing cells was examined across the length of the CeA (Supplemental Fig. 2a–x). Consistently, *Drd2* and *Drd1a* label non-overlapping populations, while *Drd2* and *Adora2a* label almost entirely overlapping populations with sparse single-labeled cells found at the far ventral portion of the CeC. At anterior positions (A/P: −0.82 to −1.2), *Drd2* strongly labels large populations within the CeC and CeL and to a lesser extent the CeM (Supplemental Fig. 2q–t). Likewise, *Drd1a* labels populations within the CeL and CeM and many fewer cells in the CeC (Supplemental Fig. 2m–p). At more posterior positions (A/P: −1.3 to −1.6) labeled cell distributions are less defined; the CeC, CeL, and CeM are sparsely labeled aside from a strongly labeled dorsal *Drd2/Adora2a* population that appears to be contiguous with the striatum (Supplemental Fig. 2l, t, x). Interestingly, the posterior CeA, especially posterior CeL, which contains the densest labeling for *Crh, Nts, Sst, Prkcd*, and *Tac2*, is only sparsely labeled with *Drd1a* (Supplemental Fig. 2p,x and Fig. 2 m, n, q, r).

To statistically assess the extent to which the *Drd2* population overlaps with markers of other identified fear-related CeA populations, co-expression of *Drd2* with *Crh, Nts, Sst, Prkcd*, and *Tac2* was quantitatively assessed across the A/P axis of the CeA (Fig. 2a–z and Supplemental Fig. 3a–j). Anterior CeA was considered to be between −0.8 and −1.2 A/P while posterior CeA was considered as between −1.3 and −1.6. Posterior to approximately −1.6 was not examined, as the CeM is absent. Positive expression within a cell was visually scored as having five or more labeled puncta within twice the diameter of the nucleus. Single-labeled images were scored then identified nuclei were overlaid and counted for none, single and double labeling.

Within the anterior CeA, *Drd2* was not found to extensively co-express with any other population examined (Fig. 2b). Within the anterior CeL and CeM *Drd2* co-expressed significantly more with *Crh* and *Tac2* respectively compared to other markers, although total co-expression was low at 19.3% and 13.1% of *Drd2* positive cells co-localized with *Crh* and *Tac2*, respectively (Fig. 2c, g, k, o, s, w and Supplemental Fig. 3c and g). Within the posterior CeC and CeL, *Drd2* co-expressed more with *Prkcd* than any other marker (36.7% and 31.5% of *Drd2* cells in the CeC and CeL, respectively); however this represented a relatively low percentage of total *Prkcd* positive cells (13.9% and 10.1% of *Prkcd* positive cells in the CeC and CeL, respectively) (Supplemental Fig. 3B). Staining for *Prkcd* was found beginning in the anterior ventral CeC forming a contiguous population to a more dorsal position posteriorly where the traditionally reported CeC and CeL population is found (Fig. 2l, m).

### Chemogenetic activation of CeA *Drd2* neurons enhances fear expression

**Fig. 3:**
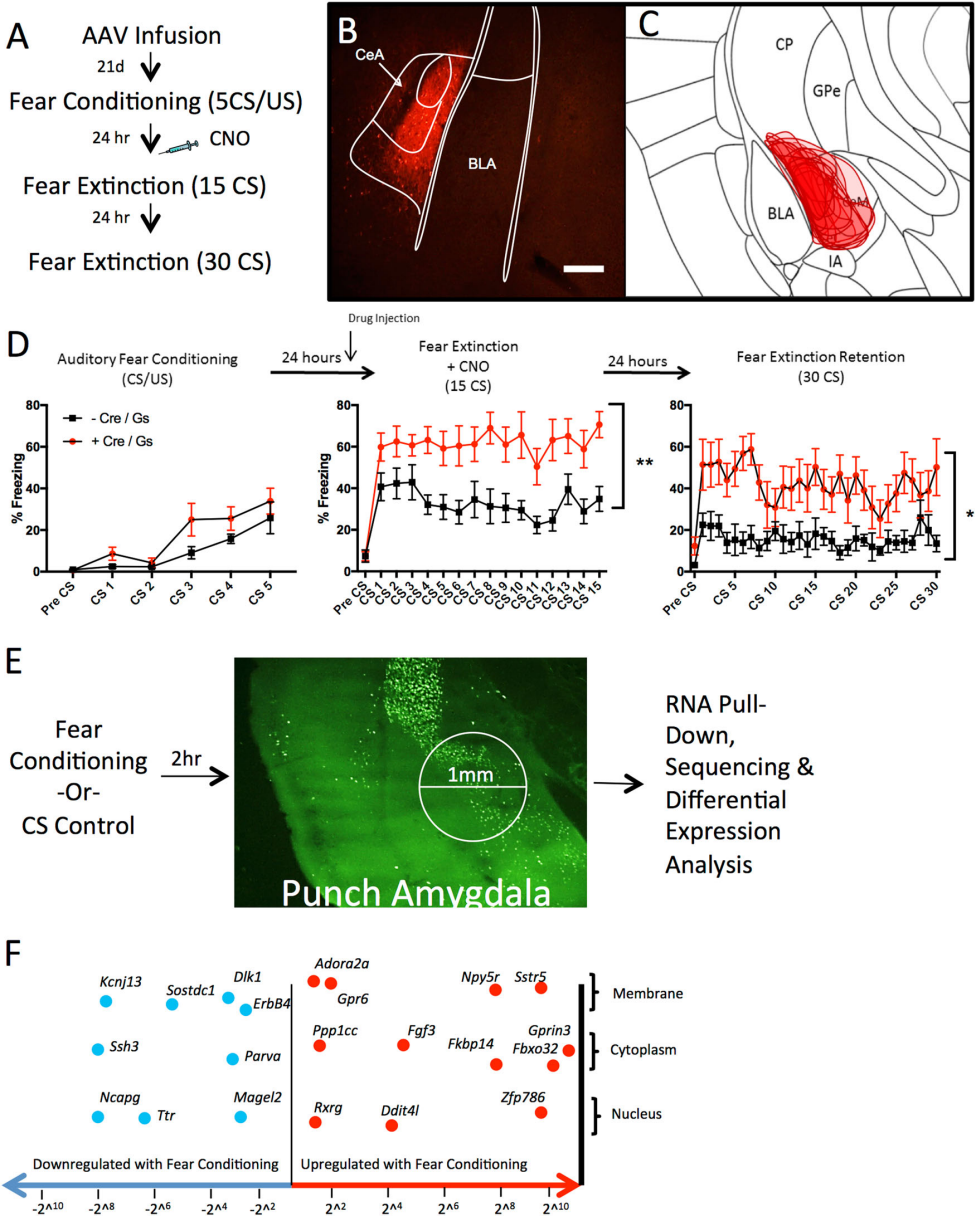
Cell-type-specific manipulation of and TRAP isolation from CeA *Drd2* population. **a** Schematic of experimental design. **b** Representative expression pattern of mCherry-tag expression in *Drd2* neurons of the amygdala. Scale bar: 200 μm. **c** Collapsed overlay of expression pattern of mCherry for Cre-expressing experimental animals. Expression is generally constrained to CeC and CeL with limited expression in CeM. **d** Mice were weakly fear conditioned to 5 CS/US pairings (6 kHz tone, 0.4 mA foot shock) (two-way RM ANOVA F(1,17) = 3.147, *p* = 0.094) (*n* = 9 DREADD and 10 Control, 1 mouse removed from DREADD group as significant outlier (Grubbs’ test)). Mice were injected i.p. with CNO 30 min prior to fear extinction session. Mice expressing Gs-DREADD-mCherry expressed significantly more fear during the entire extinction session than non-carrier controls (two-way RM ANOVA F(1,17) = 13.72, **p = 0.0018). Twenty-four hours later during the second extinction (retention) session mice expressing Gs-DREADD-mCherry expressed significantly more fear (two-way RM ANOVA F(1,17) = 11.29, *p = 0.0037). **e** Schematic of TRAP experiment. Animals were fear conditioned (5 CS/US, 0.65 mA foot shock) or exposed to training environment. Two hours later animals were sacrificed, 1 mm punches centered over CeA were taken and TRAP procedure was completed. **f** Selected differential expression results of fear conditioned vs. control animals with log-fold change on *x*-axis. Genes found to be downregulated following fear conditioning compared to controls (Blue, Leftward) include *Erbb4, Dlk1, Parva, Ssh3, Ttr*, and *Kcnj13*. Genes found to be upregulated following fear conditioning compared to controls (Red, Rightward) include *Adora2a, Gpr6, Ppp1cc, Rxrg, Fgf3, Npy5r, Sstr5, Fkbp14*, and *Gprin3*. BLA basolateral amygdala, CeC central capsular amygdala, CeL central lateral amygdala, CeM central medial amygdala, CP caudate putamen, GPe Globus Pallidus external, Intercalated Nuclei (IA). Scale bar: a–z 100 μm

To determine the precise role of the *Drd2*-expressing population in fear extinction, we directly manipulated these neurons during extinction using designer receptors exclusively activated by designer drugs (DREADDs)^38^. Drd2-Cre mice and non-Cre-expressing littermate controls were bilaterally infected with a Cre-dependent Gs-coupled DREADD virus (Fig. 3a–c)^39,40^. Gs-DREADD expression was visualized through its mCherry tag (Fig. 3b, c). Three weeks following infection, mice were mildly fear conditioned with 5 CS/US (0.4 mA US foot shock) pairings to avoid ceiling effects (Fig. 3d). A non-significant trend towards increased freezing in the Drd2-Cre mice was found during conditioning (*p* = .094); if this represents a true finding it may have been caused by leakage of the Gs-DREADD; however, freezing during the final CS/US paring was very similar between both groups (*t*-test, *p* = .43), suggesting no differences in overall fear learning. Additionally, there is no difference between groups during Pre-CS period of fear conditioning or fear extinction, suggesting no differences in baseline fear (Supplemental Fig. 4a, b). Thirty minutes prior to the extinction session (15 CS), all mice were injected with CNO (1 mg/kg, i.p. in saline). Mice that expressed Cre-recombinase and thus expressed the Gs-DREADD in *Drd2* neurons exhibited significantly more freezing to the tone throughout the extinction session. Importantly, 24 h later, after a wash-out period when DREADDs were no longer active (previous research has shown that wash-out is 6–10 h^41–43^), Gs-DREADD expressing mice again displayed significantly increased freezing to the CS compared to controls during a 30 CS extinction retention session. The rate of extinction of both groups in the initial extinction session did not significantly differ, suggesting that the enhancement in freezing during the second extinction session was likely due to blockade of extinction consolidation.

### Characterization of dynamic mRNA changes in *Drd2* cells following fear conditioning

To further characterize the *Drd2-*expressing population, we next examined expression changes in *Drd2* neurons following fear conditioning. Actively translating transcripts were examined following fear conditioning based on the expectation that active translation at this time point predicting the direction of protein expression levels 24 h later prior to fear extinction. Additionally, we expected that modulation of molecular changes precipitated by fear learning may lead to decreased fear expression or enhanced extinction. To identify actively translating mRNA transcripts, TRAP protocol was utilized^30^. The *Drd2*-Cre mouse line was crossed with the floxed-stop-TRAP (B6.129S4-Gt(ROSA)26Sortm1(CAG-EGFP/Rpl10a,-birA)Wtp/J) line to generate a double transgenic line, *Drd2*-TRAP. Expression of the L10a-GFP transgene closely recapitulated our observed expression patterns of *Drd2* (Supplemental Fig. 5)^44–46^. Next, animals were either fear conditioned (5 CS/US tone-shock pairings with 0.5 s, 0.65 mA foot shock US) or exposed to the tone CS in the chamber in the absence of any US. Fear-conditioned animals exhibited expected increases in freezing responses to the CS (Supplemental Figure 6). Animals were then sacrificed 2 h following conditioning, micropunches centered over the CeA were collected, and TRAP was performed to obtain isolated mRNA from *Drd2* neurons (Fig. 3e). High-quality RNA was retrieved from the TRAP protocol (RIN = 8.5–10). To verify the specificity of RNA pull-down, qPCR analysis of samples was performed to compare bound vs. unbound samples. Ribosomal subunit 18S was found at higher levels in the bound fraction compared to the unbound fraction, confirming enrichment for ribosomes (Supplemental Figure 7A). When expression levels of *Drd2* and *Drd1a* were compared in ribosomal bound and unbound fractions, the bound fraction had a large enrichment of *Drd2* vs. *Drd1a* transcripts when compared to the unbound fraction (Supplemental Figure 7B)^47,48^. Ribosomes specifically expressed in *Drd2* neurons were successfully pulled down and RNA collected from these pull-downs demonstrated expected characteristics of *Drd2* neurons; strong expression of *Drd2* and weak expression of *Drd1a*.

**Fig. 4:**
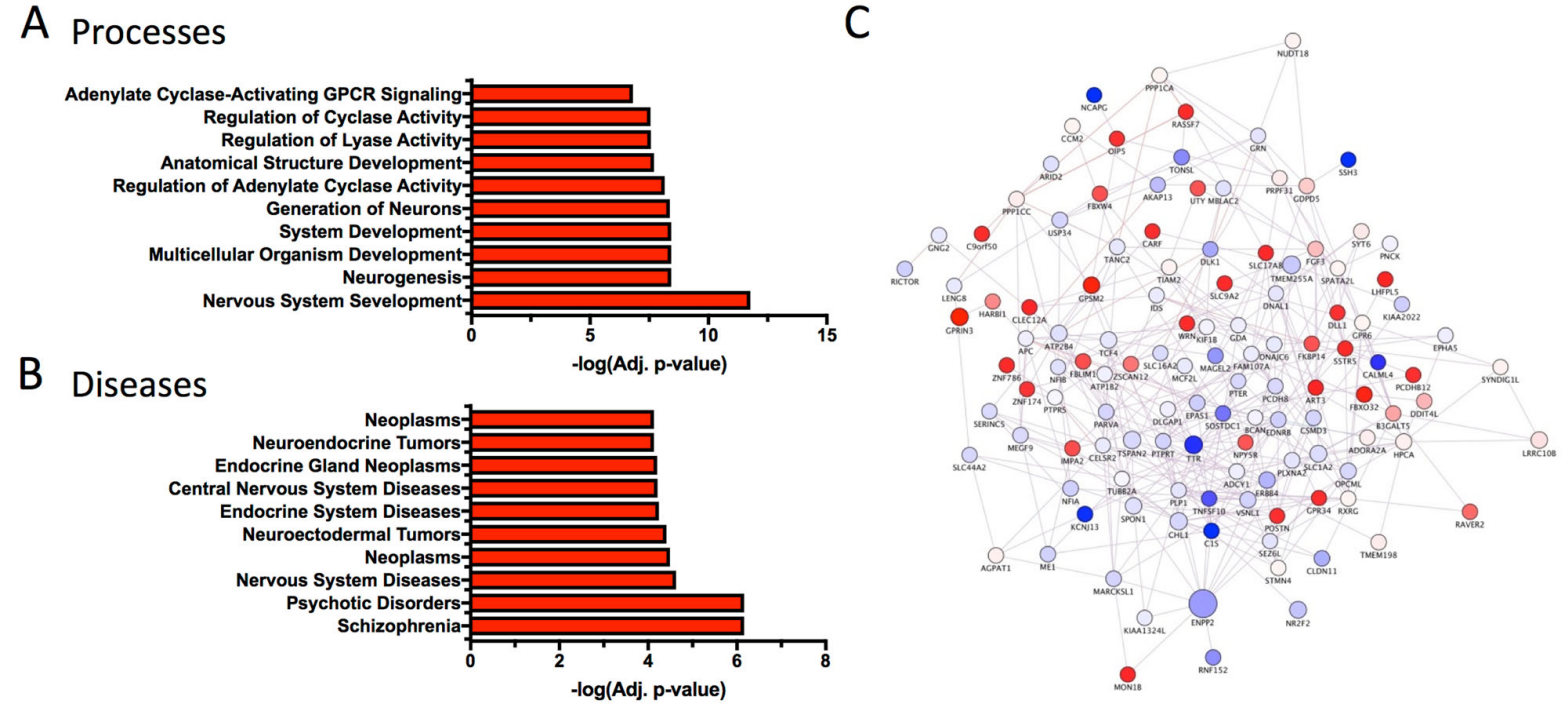
Bioinformatic analysis of differentially expressed genes in *Drd2* population following fear conditioning. **a** Enrichment analysis for the MetaCore Gene Ontology Processes identifies highly significant processes related to gene changes in *Drd2* neurons. **b** Enrichment analysis for the MetaCore Gene Ontology Diseases identifies highly significant diseases related to gene changes in *Drd2* neurons. **c** Weighted Network of genetically annotated transcripts showing differential gene expression. Differentially expressed transcripts were analyzed with the GeneMania Cytoscape plug-in using the default setting, but without extending the network with additional nodes. Genes that were not connected with others are not represented. The node size represents the −log(FDR-adjusted *p*-value), while the intensity of the color represents logFC (red nodes denote upregulation in FC, while blue nodes denote downregulation). The between-nodes edges represent relationships, the color of the edges represent the type of the relationship (76.5% co-expression in purple, 22% physical interactions in pink, 1.5% common pathway interactions in light blue), and the thickness of the edges denotes weight (i.e., strength of the pairwise relationship)

Sequencing of RNA collected from *Drd2* neuron ribosomes revealed genes dynamically regulated following fear conditioning, many of which have been previously reported to be involved in fear and anxiety-like behaviors (Fig. 3f). False discovery rate (FDR) adjusted *p*-values were calculated and FDR of 5% and fold-change of 2^0.5^ cutoffs were set (full list of differentially expressed genes is in Supplemental Table 1). Using the Mouse Gene Atlas dataset, initial analysis using Enrichr confirms amygdala specificity of pull-down and gene change (Supplemental Table 2)^49^. Further enrichment analysis using Jensen Compartments dataset confirms neuronal specificity of pull down and gene change (Supplemental Table 3). Consistent with activity dependent gene changes, MetaCore Gene Ontology Processes identifies neuronal developmental and adenylate cyclase-related processes as highly significantly recruited by fear conditioning (Fig. 4a). MetaCore Gene Ontology Diseases identifies Schizophrenia and nervous system diseases as gene categories most related to gene changes in *Drd2* neurons (Fig. 4b). Interestingly, gene set enrichment analysis (GSEA) identified gene group differences in the entire RNA-seq dataset as most concordantly similar, but in the opposite direction to two gene data sets identified in hippocampus and mPFC of humanized 22q11.2 deletion model of Schizophrenia (Supplemental Figure 8)^50,51^. This is informative for interpreting the enrichment of our top FDR-significant genes with Schizophrenia disease set by MetaCore. Weighted network analysis was completed to examine differential expression of *Drd2* genes in the context of human PTSD. Using GeneMANIA Cytoscape, differentially expressed transcripts were mapped into a self-organizing weighted network, where all of the genes were interlinked at multiple levels (co-expression, physical interactions, common pathway) (Fig. 4c)^52–54^. Overall gene network analysis reveals that differentially regulated genes are primarily co-expressed and are part of common ontologies without belonging to a single dominant pathway. To identify potential targets for pharmacological manipulation, differentially expressed genes were examined for the availability of agonists or antagonists using MetaCore Drugs for Drug targets tool (Supplemental Table 4). Finally, potential drug targets were examined in the literature for being high quality, blood–brain barrier penetrant, agonists or antagonists. Using this identification approach, inclusive of our *a priori* interest in *Adora2a* among other potential *Drd2*-neuron-specific genes (identified above), *Npy5r*, and *Rxrg* were selected for further pharmacological examination^53,55–57^. Additional markers found to be modulated with fear learning that may be of further interest also include *Sst5r, Fgf3, Erbb4, Gpr6, Fkbp14, Dlk1*, and *Ssh3*^58–65^.

Based upon the altered translational activity in *Drd2* neurons following fear conditioning, one potential route to enhance fear extinction is to pharmacologically manipulate the activity of the identified translated protein products. ADORA2A, NPY5R, and RXR were chosen as potential targets for pharmacological modulation of fear extinction, as they were robustly differentially expressed in the *Drd2* fear-regulating neuronal population, and they have well-understood mechanisms of action, making them attractive targets for pharmacological manipulation of fear extinction.

### Manipulation of ADORA2A, NPY5R, and RXR recapitulates the role of *Drd2* neurons in fear behavior

**Fig. 5:**
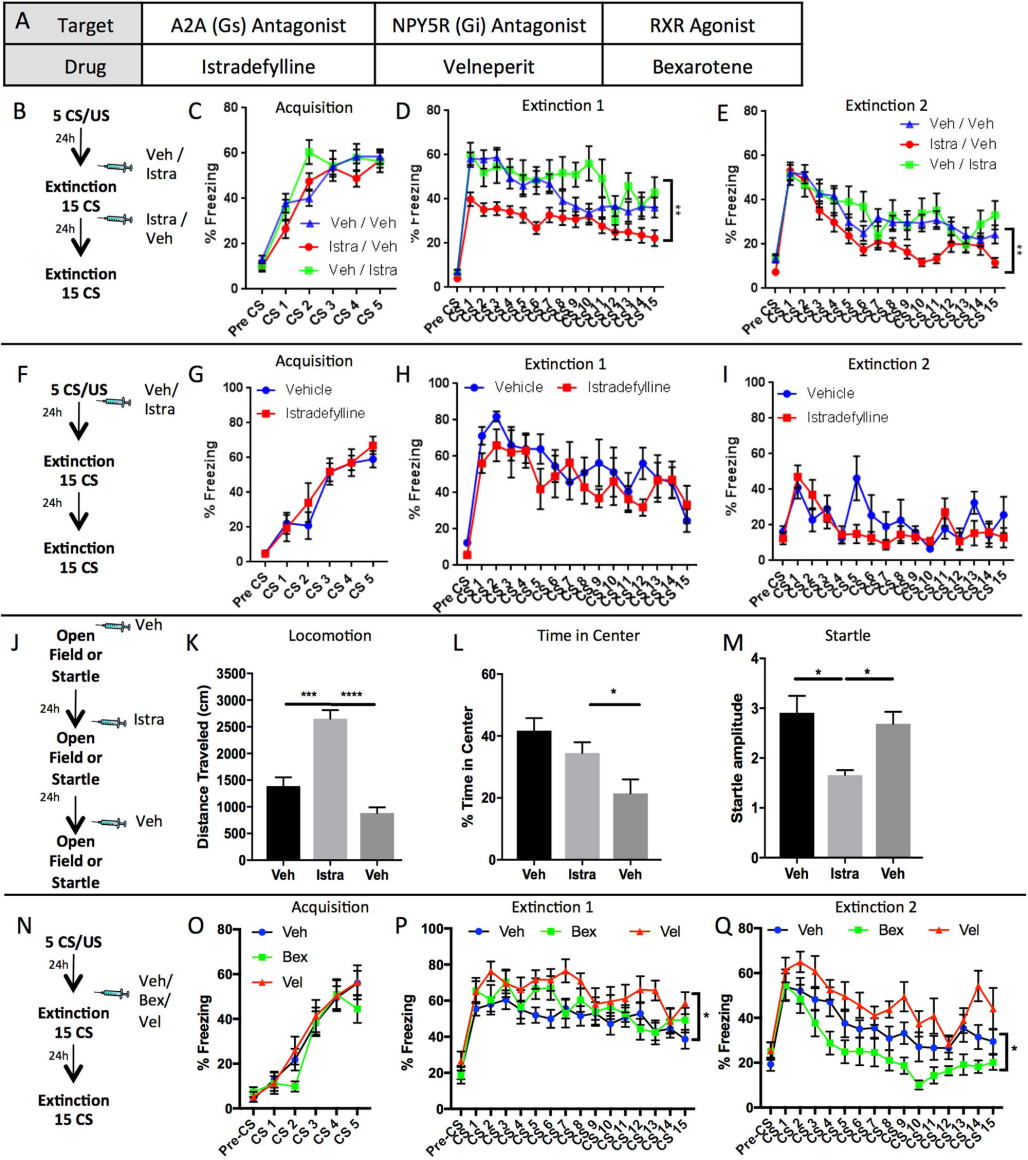
Pharmacological manipulation of ADORA2A, NPY5R, and RXR during behavior. *Adora2a, Npy5r*, and *Rxrg* were found to be increased following fear conditioning; therefore, the effect of pharmacological manipulation of ADORA2A, NPY5R, and RXR during fear extinction was examined to assess their utility as potential enhancers of exposure therapy. **a** List of pharmacological agents used, their targets and the effects of binding to target. **b** Schematic of experimental design for examination of ADORA2A antagonism by Istradefylline prior to or following fear extinction. **c** Three groups of animals were fear conditioned (5 CS/US, 0.65 mA foot shock). **d** Pre-extinction injection of Istradefylline (Istra/Veh group) causes significant decrease in freezing compared to vehicle injected controls (*n* = I/V 30, V/I 14, V/V 38) (two-way RM ANOVA F(2,71) = 10.26, *p* < .0001; Tukey’s multiple comparisons: I/V vs. V/I *p* = .0005, I/V vs. V/V *p* = .0017, V/I vs. V/V *p* = .517). **e** Animals that previously received Istradefylline prior to fear extinction (Istra/Veh) continue to express less freezing 24 h later during second extinction session (retention) compared to vehicle controls (Veh/Veh) and those that received Istradefylline following extinction (Veh/Istra) (two-way RM ANOVA F(2,69) = 5.381 (two animals removed b/c injuries from fighting, one from V/V, and one from I/V), *p* < .01; Tukey’s multiple comparisons: I/V vs. V/I *p* = .0236, I/V vs. V/V *p* = .0181, V/I vs. V/V *p* = .8988). **f** Schematic of experimental design for examination of the effect of ADORA2A antagonism following fear conditioning. **g** Two groups of mice were fear conditioned (5 CS/US, 0.65 mA foot shock) (*n* = 6 Veh, 6 Istra). **h** No effect of prior Istrafedylline treatment following fear conditioning was detected during first (two-way RM ANOVA F(1,10) = 1.22, *p* > .05) or **(i)** second extinction session (two-way RM ANOVA F(1,10) = 0.88, *p* > .05). **j** Schematic for experimental design of examination of effect of Istradefylline on locomotion, center-time, and acoustic startle. **k** Pre-session administration of vehicle (Veh) or Istradefylline (Istra). Day 2 Istradefylline treatment caused acute increase in distance traveled compared to day 1 vehicle that returned to baseline on day 3 with vehicle administration test (*n* = 6) (RM ANOVA F(1.443,8.656) = 60.77, *p* < .0001; Tukey’s Multiple Comparisons: Veh (D1) vs Istra *p* = .0009, Veh (D1) vs. Veh (D3) *p* = .0984, Veh (D3) vs. Istra *p* < .0001). **l** Pre-session administration of vehicle (Veh) or isradefylline (Istra). Day 2 Istradefylline treatment did not cause changes in anxiety-like behavior (time in center). Day 3 Veh did have reduced time in center compared to Day 2 Istra; however, this is likely due to habituation (*n* = 6) (RM ANOVA F(1.542, 9.253) = 6.602, *p* < .05; Tukey’s Multiple Comparisons Test Veh (D1) vs Istra *p* = .4570, Veh (D1) vs. Veh (D3), *p* = .0577, Veh (D3) vs. Istra, *p* = .0440). **m** Pre-session administration of vehicle (Veh) or isradefylline (Istra). Day 2 treatment with Istradefylline caused a decreased acoustic startle amplitude that did not persist into Day 3 vehicle treatment (*n* = 6) (RM ANOVA F(1.794,16.14) = 8.203, *p* = .0043; Tukey’s Multiple Comparisons Test Veh (D1) vs Istra, *p* = .0205, Veh (D1) vs. Veh (D3), *p* = .7924, Veh (D3) vs. Istra *p* = .0111)). **n** Schematic for experimental design of examination of effects of Venelperit and Bexarotene. **o** Cohorts of mice were fear conditioned (5 CS/US, 0.65 mA foot shock) (*n* = 20 Veh, 12 Vel, 12 Bex), no differences between groups was detected. **p** Pre-extinction injection of Velneperit caused increased freezing when compared to vehicle group. No difference between Bexarotene and vehicle group was detected (two-way RM ANOVA, F (2,41) = 3.325, *p* < .05, Dunnett’s Multiple Comparisons Test: Veh vs. Bex, *p* = .5712, Veh vs. Vel, *p* = .0255). **q** The next day, 24 h later, during a second extinction session, significant main effect of treatment was detected (two-way RM ANOVA, F (2,41) = 8.52, *p* < .001). Injection of Velneperit prior to first extinction session did not cause significant changes in behavior compared to vehicle; however, prior injection of Bexarotene caused a significant reduction in freezing (Dunnett’s Multiple Comparisons Test: Veh vs. Bex, *p* = .0148, Veh vs. Vel, *p* = .1491)

Agonists and antagonists targeting ADORA2A, NPY5R, and RXR receptors were chosen based on prior studies from the literature (Fig. 5a)^66^. *Adora2a* was an attractive candidate for further inquiry and was chosen for initial characterization based on a number of reasons: (1) it has previously been shown to almost entirely co-express with *Drd2* (Fig. 1g–l) within the amygdala, and (2) several pharmacological agents targeting ADORA2A are currently in clinical trials or have been approved for use in humans^67,68^. The highly selective ADORA2A antagonist, Istradefylline, is selective for ADORA2A over ADORA1 with a Ki of 2.2 and 150 nM, respectively^69,70^.

To examine the effect of ADORA2A antagonism on fear extinction three cohorts of mice were fear conditioned (5 CS/US, 0.65 mA foot shock) (Fig. 5b, c). Twenty-four hours following fear conditioning, and 30 min prior to fear extinction, mice were injected with Istradefylline (3 mg/kg^71^) (group I/V) or vehicle (10% DMSO, 1% NP-40 in saline i.p.) (groups V/V and V/I) (Fig. 5b). Additionally, immediately following fear extinction (15 CS) mice were injected with Istradefylline (3 mg/kg) (group V/I) or vehicle (groups I/V and V/V). Injection of Istradefylline, but not vehicle prior to fear extinction (15 CS, Extinction 1) greatly decreased freezing during extinction training when drug was on-board (Fig. 5d). Twenty-four hours later, following drug clearance, mice that had previously been injected with Istradefylline prior to fear extinction, but not those injected following it, expressed significantly less freezing during a second extinction session (15CS, Extinction 2) (Fig. 5e). Replication data in a separate cohort of mice may be found in Supplemental Figure 9. These data suggest that blockade of ADORA2A during fear extinction, but not during extinction consolidation, is sufficient to enhance fear extinction learning.

To further examine the role of ADORA2A in fear consolidation, a separate cohort of mice was fear conditioned (5 CS/US, 0.65 mA) and given injections of Istradefylline (3 mg/kg, i.p.) or vehicle directly following the fear conditioning training session (5 CS/US, 0.65 mA foot shock), (Fig. 5f, g). During subsequent extinction sessions, 24 and 48 h later, no significant effect of ADORA2A blockade on fear consolidation was detected (Fig. 5h, i).

Istradefylline is a potential drug treatment for Parkinson’s disease, thus it is possible that locomotor effects obscured the effects of drug on fear behavior; therefore, two separate cohorts of mice were tested for locomotor and anxiety-like behaviors in an open field, and acoustic startle responses on consecutive days (Fig. 5j). Injection of Istradefylline (3 mg/kg) but not vehicle significantly increased the distance traveled in the open field; however 24 h later the distance traveled had returned to pre-treatment levels (Fig. 5k). Importantly, increased distance traveled was not accompanied by any anxiogenic or anxiolytic effects in the open field (Fig. 5l). Decreased time in center across days is likely due to habituation to the chamber context. Finally, Istradefylline acutely decreased baseline acoustic startle; however this effect was not present 24 h later when startle amplitude returned to pre-treatment levels (Fig. 5m). Together, these data suggest that the effects of Istradefylline in enhancing extinction retention tested 24 h after drug administration are unlikely the result of alterations in locomotion or effects on anxiety-like behavior, per se.

NPY5R and RXR were additional identified targets that were examined for pharmacological enhancement of fear extinction. Velneperit antagonizes NPY5R, while Bexarotene is a RXR agonist (Fig. 5a, n). Three cohorts of animals were fear conditioned (5 CS/US, 0.65 mA foot shock) (Fig. 5o). Twenty-four hours later, 90 min prior to fear extinction (15 CS), animals were given injections of Velneperit (NPY5R Antagonist, 100 mg/kg^72–74^), Bexarotene (RXR agonist, 50 mg/kg^57^) or vehicle (DMSO). Animals treated with Velneperit (NPY5R antagonist) expressed significantly more freezing than animals injected with vehicle. No differences between Bexarotene injected and vehicle injected groups were detected (Fig. 5p). 24 h later during a second extinction session (15 CS) in the absence of any drug, no difference between Velneperit and vehicle groups was detected. During the same test session animals previously treated with Bexarotene prior to the first extinction session expressed significantly reduced freezing compared with controls (Fig. 5q). Replication data may be found in Supplemental Figure 10. These pharmacological agents predictably affected fear extinction learning in a manner consistent with our hypothesized role for the *Drd2* population being a fear-supporting population whose activation or inhibition is sufficient to modulate fear. Antagonizing G_αs_-coupled ADORA2A dramatically decreased fear expression, as would be expected by decreasing activity of a fear-supporting population. In contrast, antagonizing G_αi_-coupled NPY5R increased fear expression as would be expected by decreasing inhibition of (increasing activity of) a fear-supporting population. Activation of RXR may act to generally enhance extinction consolidation as observed with Bexarotene treatment, although the mechanism by which this may occur is unclear as RXRs are nuclear hormone receptors with a variety of binding partners^75^.

### Dynamic regulation of *Drd2* after fear extinction

*Drd2* expression was not significantly changed after fear conditioning in the above reported TRAP study; however, the literature suggests that D2R is involved in the control and consolidation of fear and extinction learning. Therefore, the dynamic regulation of *Drd2* was examined after fear extinction. Two groups (FC 1 and FC 30) of animals were fear conditioned (5 CS/US, 0.65 mA foot shock) (Supplemental Figure 11A). 24 h later three groups received differing CS exposures; FC30 received 30 CS presentations; FC1 received 1 CS presentation and remained in the chamber for the remainder of the session; HC30 received exposure to 30 CS presentations with no previous training experience (Supplemental Figure 11B). A home cage (HC) control group was also included. Each cohort of mice was sacrificed 2 h following extinction training, RNA was isolated from 1 mm micropunch centered over the CeA, and *Drd2* expression levels were examined via qPCR. *Drd2* mRNA expression was significantly increased in the extinction group (FC30) when compared to all other groups and no significant change from HC was found in either HC30 or FC1 groups (Supplemental Figure 11C). These data suggest that dynamic regulation of *Drd2* may be involved in the consolidation of fear extinction, potentially increasing inhibition of this population through G_αi_ coupled D2 receptors. This dynamic regulation with fear extinction consolidation is consistent with our findings of differential modulation of extinction learning with targeted *Drd2*-cell-type-specific pharmacological approaches.

## Discussion

The present study: (1) examined the distribution and co-expression of *Drd2* with *Drd1a, Adora2a, Crh, Nts, Sst, Prkcd*, and *Tac2* across the A/P axis; (2) identified *Drd2* expressing neurons as a fear-supporting population through direct chemogenetic manipulation; (3) characterized cell-type-specific translational changes following fear conditioning and identified many dynamically regulated genes including *Adora2a, Npy5r, Rxrg, Sst5r, Fgf3, Erbb4, Fkbp14, Dlk1*, and *Ssh3*; and finally, (4) pharmacologically manipulated ADORA2A, NPY5R, and RXR to assess their viability as potential *Drd2* cell-type-specific targets for pharmacological enhancement of fear extinction.

The identification of a fear-supporting population in the CeC is consistent with previous findings that the CeC specifically receives input from the fear promoting prelimbic cortex as well as other anxiety and pain related areas^76,77^. Our findings of strong co-expression of *Drd2* and *Adora2a* but not *Drd1a* are consistent with findings in other regions^48^. Interestingly, we found lower co-expression of *Drd2* with *Prkcd* in the posterior CeL compared to reports by De Bundel et al. and in the anterior CeC compared to reports by Kim et al.^19,78^. The former instance is explained by De Bundel’s use of a Drd2::GFP reporter mouse; reporter mice may strongly express a transgene in cells that only express lower levels of the native transcript and thus were below our detection criteria. Likewise, discrepancies with Kim et al. are likely due to our use of stricter criteria for positive expression. In either case, data from both reports support our findings of *Drd2* as a fear-supporting population. Another interesting discrepancy between our data and those reported by Kim et al. is that we found less *Drd1a* expression in the posterior CeL. This is remarkable because the posterior CeL contains the densest *Crh, Nts, Sst, Prkcd*, and *Tac2*, populations that were reported to correspond with *Drd1a* neurons in this area. This discrepancy may again be due to our more strict criteria for positively expressing cells.

Overall, the presented behavioral data are remarkably consistent across experiments. Manipulation of the *Drd2* neuronal population either through Gs–DREADD, or the inhibition of ADORA2A (G_αs_) or NPY5R (G_αi_), drives fear expression in directions consistent with this being a fear supporting population. ADORA2a is known to be co-expressed with D2R and these receptors have been shown to have opposing actions, suggesting that both receptors may be viable candidates for modulation of a single sub-population^48,79^. An important consideration is that drugs were administered systemically, thus making it impossible to claim that effects were mediated exclusively through receptors found in the CeA. However, as the goal of this line of research is to identify potentially clinically relevant targets for enhancement of therapy, it is advantageous to test candidates as they would be used in the clinic, that is, systemically and prior to exposure therapy.

The finding of an acute increase in locomotion with global A_2A_R antagonism is consistent with reports in the literature and is expected because manipulation of the indirect pathway is a common treatment for Parkinson’s Disease^27,79^. Additionally, locomotor effects observed with the ADORA2A antagonist closely mirror results observed from direct DREADD manipulation of *Adora2a* neurons^80^. The transience of locomotor effects as well as the absence of effects on anxiety-like behavior suggest that changes in freezing during subsequent testing are due to effects on learning and are not the result of locomotor changes. These results are also consistent with reports that ADORA2A antagonism with SCH58261 results in deficits in contextual fear conditioning^81^.

Profiling changes in actively translating RNAs using TRAP protocols provides a unique window into the acute responses of these neurons to a learning event. We sought to identify transcripts that were differentially regulated following fear learning, so that changes in protein activity might be pharmacologically opposed at a later time point; during fear extinction. There are several other important time points to compare including prior to and following fear extinction, which will be important subjects for future investigation. Additionally, although tone-alone control was the best available control condition to address gene expression differences as a function of tone-shock learning, additional controls such as shock alone or unpaired tone-shock may yield important information about the specificity off this manipulation to associative learning.

Bioinformatic analysis of TRAP-seq data emphatically confirms specificity of pull-down to amygdala neurons. Network analysis reveals that identified differentially expressed genes are primarily co-expressed. Although genes do not to a great extent belong to a single pathway, they are part of common ontologies suggesting domains of proteins that may be valuable to interrogate in the future. Several genes including *Adora2a, Sst5r, Npy5r, Fgf3*, and *Erbb4*, have been directly implicated in or are in well-established signaling pathways implicated in the control of fear learning. Others genes such as *Rxrg, Gpr6, Fkbp14, Parva, Dlk1*, and *Ssh3* have not been studied in the context of fear biology, but may provide valuable insights upon further investigation. Interestingly, several of these genes, most prominently *Adora2a* and *SstR5*, have been implicated in human anxiety disorders^82,83^.

Data presented here identifies potential pharmacological enhancers of extinction by leveraging cell-type-specific techniques in a fear-controlling population. This approach represents a potential avenue for predicting novel targets for the modulation of emotional learning, generating more specific and effective treatments for psychiatric disorders such as PTSD.

## Methods

### Animals

C57BL/6J mice were obtained from Jackson Laboratories (Bar Harbor, ME). B6.FVB(Cg)-Tg(Drd2-Cre)ER43Gsat/Mmucd mice were obtained from the MMRRC and produced as part of the GENSAT BAC Transgenic Project. Rosa26 fs-TRAP (B6.129S4-Gt(ROSA)26Sortm1(CAG-EGFP/Rpl10a,-birA)Wtp/J) mice were obtained from Jackson Laboratories. *Drd2*-TRAP mice were generated by crossing *Drd2*-Cre and Rosa26 fs-TRAP lines. All mice were adult (8–12 weeks) at the time of behavioral training. All mice were group housed and maintained on a 12 h:12 h light: dark cycle. Mice were housed in a temperature-controlled colony and given unrestricted access to food and water. All procedures conformed to National Institutes of Health guidelines and were approved by Emory University Institutional Animal Care and use Committee. Animal numbers were calculated using G*Power 3 software using previous experiments to inform expected means and standard deviations for expected large and medium effect sizes for chemogenetic and pharmacological manipulations, respectively. Animals were assigned to groups based upon genotype or randomized to treatment. Experimenter was blinded to genotype of animals. Blinding to drug administration was not possible; however, animal IDs were coded during data analysis.

### Surgical procedures

Mice were deeply anesthetized with a Ketamine/Dexdormitor (medetomidine) mixture and their heads fixed into a stereotaxic instrument (Kopf Instruments). Stereotaxic coordinates were identified from Paxinos and Franklin^84^ and heads were leveled using lambda and bregma. For viral delivery (Fig. 3), a 10 μl microsyringe (Hamilton) was lowered to coordinates just above CeA (A/P −1.2, M/L ± 3.0, D/V −4.8) and 0.5 μl of AAV_5_-hSyn-DIO-rM3D(Gs)-mCherry (UNC Viral Vector Core) was infused at 0.1 μl/min using a microsyringe pump. After infusion, syringes remained in place for 15 min before being slowly withdrawn. After bilateral infusion, incisions were sutured closed using nylon monofilament (Ethicon). For all surgeries, body temperature was maintained using a heating pad. After completion of surgery, anesthesia was reversed using Antisedan (atipamezole) and mice were allowed to recover on heating pads.

### Drug administration

Clozapine-*N*-Oxide (Sigma) was diluted in sterile saline and administered at 1 mg/kg i.p. 30 min prior to behavioral testing. Istradefylline (Tocris # 5417) was dissolved in DMSO and diluted to 10% DMSO, 1% NP-40 in sterile saline immediately prior to i.p. administration at 3 mg/kg. Velneperit (MEdChem Express #342577-38-2) has very low solubility in water, thus it was dissolved in pure DMSO prior to injection and injected at 100 mg/kg in 0.03 ml using an insulin syringe. Bexarotene (Tocris # 5819) also has limited solubility in water, thus it was dissolved in pure DMSO prior to injection and injected i.p. at 50 mg/kg in a volume of 0.03 ml using an insulin syringe. Control animals received equal volumes of vehicle. These volumes of pure DMSO have been previously tested and validated to cause no adverse health effects in adult mice.

### Behavioral assays

#### Auditory cue-dependent fear conditioning

Mice were habituated to fear-conditioning chambers (Med Associates Inc., St Albans, VT) for 10 min each of 2 days prior to fear conditioning. Mice were conditioned to five tones (30 s, 6 kHz, 65–70 db) co-terminating with a 1 s foot shock (0.65 mA, 1 mA for *Drd2* expression experiment, or 0.4 mA for mild conditioning).

#### Auditory cue-dependent extinction

Cue-dependent fear extinction was tested 24 h after fear conditioning and extinction retention occurred 24 h after fear expression. For extinction, mice were placed in a novel context with a different olfactory cue, lighting and flooring and exposed to 15 or 30  tones (30 s, 6 kHz, 65–70 db) with an inter-trial-interval of 60 s. Freezing was measured using Freeze View software (Coulbourn Instruments Inc., Whitehall, PA).

#### Open Field

Open field chambers (Med Associates) were placed in a dimly lit room. Mice were placed in the chamber for 10 min and allowed to explore.

#### Brain collection following behavior for qPCR analysis of Drd2

Examination of changes in *Drd2* expression following behavioral experiments included four groups: (1) a Home Cage (HC) control group that remained undisturbed in their home cage throughout the experiment; (2) the primary experimental group (FC30), which received fear conditioning and extinction (30 CS) as described above; (3) a tone-alone control group (HC30) that remained in the home cage during training but was exposed to the same 30 tone presentations as the FC30 group in the extinction context; (4) a conditioned control group (FC1) that was fear conditioned as in the FC30 group but only exposed to one tone 24 h later. Brains were extracted 2 h after fear extinction or tone exposure. Brains from HC control animals were also extracted during this time.

#### Real-time PCR

RNA was reverse transcribed using SuperScript 4 (Invitrogen). Quantitative PCR was performed on cDNA with each sample run in triplicate technical replicates. Reactions contained 12 μl Taqman Gene Expression Master Mix (Applied Biosystems), 1 μl of forward and reverse primer, 1 μl of 5 ng/μl cDNA, and 6 μl water. Primers were proprietary FAM-labeled probes from Life Technologies. Quantification of qPCR was performed on Applied Biosystems 7500 Real-Time PCR System. Cycling parameters were 10 min at 95 °C, 40 cycles of amplification of 15 s at 95 °C and 60 s at 60 °C, and a dissociation step of 15 s 95 °C, 60 s at 60 °C, 15 s 95 °C. Fold changes were calculated as ΔΔCT values normalized to levels of GAPDH or 18S mRNA. Values presented as fold change ± s.e.m.

#### RNA-seq library preparation

Libraries were generated from 1 ng of Total RNA using the SMARTer HV kit (Clonetech), barcoding and sequencing primers were added using NexteraXT DNA kit. Libraries were validated by microelectrophoresis, quantified, pooled and clustered on Illumina TruSeq v3 flowcell. Clustered flowcell was sequenced on an Illumina HiSeq 1000 in 50-base paired end reactions. Approximately 25 million sequencing reads were collected per sample.

#### Analysis of RNA-sequencing data

RNA-sequencing data was analyzed using Tuxedo DESeq analysis software. Differential expression between HC and FC groups were obtained and used for further analysis. Using the *q* value of <0.05 as a cutoff, only highly significant returns were used for further analysis. To ensure that genes had a large enough difference in expression to warrant pharmacological manipulation, only those with differences in expression greater than 2^0.5^ or ~141% were considered.

#### Bioinformatics

Enrichment analysis for Mouse Gene Atlas dataset and Jensen Compartments, was performed with Enrichr^49^.

Enrichment analysis for Gene Ontology Processes and Diseases was performed using MetaCore (Clarivate) Gene Set Enrichment Analysis was used to identify gene with concordant directional effects^51^. Weighted gene network analysis was performed using GeneMania at the default setting^52^. Network data are presented in Dataset Fig. 4c and Supplemental Tables 2 and 3 and were visualized in Cytoscape^85^ and presented in Fig. 4. Next using the MetaCore “Drugs for Drug targets” ‘Drug Gene Interaction Database’ (http://www.dgidb.org/) returns were examined for having a known pharmacological agent that modifies its activity. Genes lacking viable pharmacological modulators were eliminated. Sequencing data including fasq files available through the NCBI gene expression omnibus accession number GSE114784.

#### Translating ribosome affinity purification (TRAP)

TRAP procedure was completed as described in Heintz and colleagues^30^. Adult *Drd2*-TRAP mice were anesthetized; their brains removed and snap-frozen. Bilateral 1 mm punches were collected and pooled from three animals per sample (*n* = 2 (HC) and *n* = 3 (FC)). Messenger RNA was isolated from eGFP-tagged ribosomes, as described in ref. ^30^. RNA was assessed for quality using the Bioanalyzer Pico (Agilent, Santa Clara, CA). All samples returned RINs (RNA Integrity Numbers) of 8.5 or greater.

### Statistics

Statistical analyses were performed using Prism 6 or 7 by Graph Pad. All data is presented as mean + /- s.e.m. Homogeneity of variances was tested using the Bartlett’s test. Fear extinction experiments were examined using a repeated-measures ANOVA with drug as the between-subjects factor and tone presentation as the within subject factor. Open field activity or acoustic startle for Istrafefylline experiments was compared using a repeated measures ANOVA and a Tukey’s multiple comparisons analysis. For qPCR delta delta CTs of data were compared by Student’s *t*-test between bound and unbound fractions. For all tests statistical significance was set at *p* < .05. For quantification of FISH RNA-Scope results, numbers of expressing verses co-expressing cells were compared non-paprametric using Mann–Whitney’s test. Outliers were tested for using Grubb’s Outlier test, only 1 significant outlier was removed (noted in figure legend).

#### FISH - RNAscope staining

For in situ analysis of *Drd2* co-localization of different amygdala markers adult male C57BL/6J mice were obtained from Jackson Laboratories. All mice were sacrificed at the same time.

Staining for RNA of interest was accomplished using RNA Scope Fluorescent Multiplex 2.5 labeling kit (ACD Bio). Probes utilized for staining are: mm-Nts-C1, mm-Nts-C2, mm-Tac2-C2, mm-Sst-C1, mm-Sst-C2, mm-Crh-C1, mm, Prkcd-C1, mm-Prkcd-C3, mm-Drd2-C3, mm-Dkk3-C1, mm-Drd1a-C2, and mm-Adora2a-C1. Brains were extracted and snap-frozen in methyl-butane on dry ice. Sections were taken at a width of 16 µm. RNAscope procedures were completed to manufacturers’ specifications (ACD Bio).

#### Quantitative in situ analysis

Tissue was obtained and stained from each of five adult male mice. Amygdalae were analyzed bilaterally for each pair of in situ probes leading to a minimum of *n* = 8 amygdala analyzed for each probe combination. Cells were identified as expressing an mRNA when five or the equivalent area of five or more fluorescent puncta could be identified within twice the diameter of the nucleus centered over the nucleus.

#### Image acquisition

Images were acquired with the experimenter blinded to the probes used. 16-bit images of staining were acquired on a Leica SP8 confocal microscope using a ×10, ×20, or ×40 objective. Images for Figs. 1a–f and 4b, e were acquired using a Zeiss Imager a1 with a ×2 or ×4 objective. Within a sample, images used for quantification were acquired with identical settings for laser power, detector gain, and amplifier offset. Images were acquired as a z-stack of 10 steps of 0.5 µm each. Max intensity projections were then created and analyzed.

## Electronic supplementary material

Supplemental Figure 1

Supplemental Figure 2

Supplemental Figure 3

Supplemental Figure 4

Supplemental Figure 5

Supplemental Figure 6

Supplemental Figure 7

Supplemental Figure 8

Supplemental Figure 9

Supplemental Figure 10

Supplemental Figure 11

Supplemental Table 1

Supplemental Tables 2 and 3

Supplemental Table 4a

Supplemental Table 4b

Supplemental Table 4c

Supplemental Table 4d

Supplemental Figure Legends

## References

[1] Davis M, Walker DL, Myers KM (2003). Role of the amygdala in fear extinction measured with potentiated startle. Ann. N. Y. Acad. Sci..

[2] Pare D, Quirk GJ, Ledoux JE (2004). New vistas on amygdala networks in conditioned fear. J. Neurophysiol..

[3] McDONALD AJ (1982). Cytoarchitecture of the central amygdaloid nucleus of the rat. J. Comp. Neurol..

[4] Hitchcock JM, Sananes CB, Davis M (1989). Sensitization of the startle reflex by footshock: blockade by lesions of the central nucleus of the amygdala or its efferent pathway to the brainstem. Behav. Neurosci..

[5] Ciocchi S (2010). Encoding of conditioned fear in central amygdala inhibitory circuits. Nature.

[6] McCullough K, Morrison F, Ressler K (2016). Bridging the gap: towards a cell-type specific understanding of neural circuits underlying fear behaviors. Neurobiol. Learn. Mem..

[7] Haubensak W (2010). Genetic dissection of an amygdala microcircuit that gates conditioned fear. Nature.

[8] Andero R, Dias BG, Ressler KJ (2014). A role for Tac2, NkB, and Nk3 receptor in normal and dysregulated fear memory consolidation. Neuron.

[9] Li H (2013). Experience-dependent modification of a central amygdala fear circuit. Nat. Neurosci..

[10] McCall JG (2015). CRH engagement of the locus coeruleus noradrenergic system mediates stress-induced anxiety. Neuron.

[11] Myers KM, Davis M (2007). Mechanisms of fear extinction. Mol. Psychiatry.

[12] Maren S, Fanselow MS (1996). The amygdala and fear conditioning: has the nut been cracked?. Neuron.

[13] Maren S, Holmes A (2016). Stress and fear extinction. Neuropsychopharmacology.

[14] Rothbaum BO (2014). A randomized, double-blind evaluation of D-cycloserine or alprazolam combined with virtual reality exposure therapy for posttraumatic stress disorder in Iraq and Afghanistan War veterans. Am. J. Psychiatry.

[15] McDONALD AJ (2003). Is there an amygdala and how far does it extend?. Ann. N. Y. Acad. Sci..

[16] Smith Y, Bevan M, Shink E, Bolam J (1998). Microcircuitry of the direct and indirect pathways of the basal ganglia. Neuroscience.

[17] Pollack AE (2001). Anatomy, physiology, and pharmacology of the basal ganglia. Neurol. Clin..

[18] Fadok JP (2017). A competitive inhibitory circuit for selection of active and passive fear responses. Nature.

[19] Kim J, Zhang X, Muralidhar S, LeBlanc SA, Tonegawa S (2017). Basolateral to central amygdala neural circuits for appetitive behaviors. Neuron.

[20] Han S, Soleiman MT, Soden ME, Zweifel LS, Palmiter RD (2015). Elucidating an affective pain circuit that creates a threat memory. Cell.

[21] de la Mora MP, Gallegos-Cari A, Arizmendi-García Y, Marcellino D, Fuxe K (2010). Role of dopamine receptor mechanisms in the amygdaloid modulation of fear and anxiety: structural and functional analysis. Prog. Neurobiol..

[22] Abraham AD, Neve KA, Lattal KM (2014). Dopamine and extinction: a convergence of theory with fear and reward circuitry. Neurobiol. Learn. Mem..

[23] Fernández RS, Boccia MM, Pedreira ME (2016). The fate of memory: reconsolidation and the case of prediction error. Neurosci. Biobehav. Rev..

[24] Li L (2016). The association between genetic variants in the dopaminergic system and posttraumatic stress disorder: a meta-analysis. Medicine.

[25] Barch, D. M., Pagliaccio, D. & Luking, K. *Behavioral Neuroscience of Motivation* 411–449 (Springer International Publishing, Switzerland, 2016).

[26] Lenka A, Arumugham SS, Christopher R, Pal PK (2016). Genetic substrates of psychosis in patients with Parkinson’s disease: a critical review. J. Neurol. Sci..

[27] Ponnusamy R, Nissim HA, Barad M (2005). Systemic blockade of D2-like dopamine receptors facilitates extinction of conditioned fear in mice. Learn. Mem..

[28] Mora DeLa (2012). Distribution of dopamine D2-like receptors in the rat amygdala and their role in the modulation of unconditioned fear and anxiety. Neuroscience.

[29] Guarraci FA, Frohardt RJ, Falls WA, Kapp BS (2000). The effects of intra-amygdaloid infusions of a D_2_ dopamine receptor antagonist on Pavlovian fear conditioning. Behav. Neurosci..

[30] Heiman M, Kulicke R, Fenster RJ, Greengard P, Heintz N (2014). Cell type–specific mRNA purification by translating ribosome affinity purification (TRAP). Nat. Protoc..

[31] Heiman M (2008). A translational profiling approach for the molecular characterization of CNS cell types. Cell.

[32] Sunkin SM (2012). Allen Brain Atlas: an integrated spatio-temporal portal for exploring the central nervous system. Nucleic Acids Res..

[33] Paxinos G (2013). Paxinos and Franklin’s the Mouse Brain in Stereotaxic Coordinates.

[34] McCullough KM (2016). Molecular characterization of Thy1 expressing fear-inhibiting neurons within the basolateral amygdala. Nat. Commun..

[35] Mo A (2015). Epigenomic signatures of neuronal diversity in the mammalian brain. Neuron.

[36] Bourgeais L, Gauriau C, Bernard JF (2001). Projections from the nociceptive area of the central nucleus of the amygdala to the forebrain: a PHA-L study in the rat. Eur. J. Neurosci..

[37] Yu K, Garcia da Silva P, Albeanu DF, Li B (2016). Central amygdala somatostatin neurons gate passive and active defensive behaviors. J. Neurosci..

[38] Rogan SC, Roth BL (2011). Remote control of neuronal signaling. Pharmacol. Rev..

[39] Maze I (2014). G9a influences neuronal subtype specification in striatum. Nat. Neurosci..

[40] Lobo MK (2013). ΔFosB induction in striatal medium spiny neuron subtypes in response to chronic pharmacological, emotional, and optogenetic stimuli. J. Neurosci..

[41] Wess J, Nakajima K, Jain S (2013). Novel designer receptors to probe GPCR signaling and physiology. Trends Pharmacol. Sci..

[42] Guettier JM (2009). A chemical-genetic approach to study G protein regulation of beta cell function in vivo. Proc. Natl Acad. Sci. USA.

[43] Alexander GM (2009). Remote control of neuronal activity in transgenic mice expressing evolved G protein-coupled receptors. Neuron.

[44] Gangarossa G (2013). Spatial distribution of D1R- and D2R-expressing medium-sized spiny neurons differs along the rostro-caudal axis of the mouse dorsal striatum. Front. Neural Circuits.

[45] Gangarossa G (2012). Characterization of dopamine D1 and D2 receptor-expressing neurons in the mouse hippocampus. Hippocampus.

[46] Gong S (2007). Targeting Cre recombinase to specific neuron populations with bacterial artificial chromosome constructs. J. Neurosci..

[47] Le Moine C, Bloch B (1995). D1 and D2 dopamine receptor gene expression in the rat striatum: sensitive cRNA probes demonstrate prominent segregation of D1 and D2 mRNAs in distinct neuronal populations of the dorsal and ventral striatum. J. Comp. Neurol..

[48] Oude-Ophuis RJ, Boender AJ, van Rozen R, Adan RA (2014). Cannabinoid, melanocortin and opioid receptor expression on DRD1 and DRD2 subpopulations in rat striatum. Front. Neuroanat..

[49] Chen EY (2013). Enrichr: interactive and collaborative HTML5 gene list enrichment analysis tool. BMC Bioinformatics.

[50] Stark KL (2008). Altered brain microRNA biogenesis contributes to phenotypic deficits in a 22q11-deletion mouse model. Nat. Genet..

[51] Subramanian A (2005). Gene set enrichment analysis: a knowledge-based approach for interpreting genome-wide expression profiles. Proc. Natl Acad. Sci. USA.

[52] Warde-Farley D (2010). The GeneMANIA prediction server: biological network integration for gene prioritization and predicting gene function. Nucleic Acids Res..

[53] Mouro FM (2017). Chronic and acute adenosine A2A receptor blockade prevents long-term episodic memory disruption caused by acute cannabinoid CB1 receptor activation. Neuropharmacology.

[54] Kramer A, Green J, Pollard J, Tugendreich S (2014). Causal analysis approaches in ingenuity pathway analysis. Bioinformatics.

[55] Yukioka H (2010). [A potent and selective neuropeptide Y Y5-receptor antagonist, S-2367, as an anti-obesity agent]. *Nihon yakurigaku zasshi*. Folia Pharmacol. Jpn..

[56] Bhat SP, Sharma A (2017). Current drug targets in obesity pharmacotherapy - a review. Curr. Drug. Targets.

[57] Wang S, Wen P, Wood S (2016). Effect of LXR/RXR agonism on brain and CSF Abeta40 levels in rats. F1000Res..

[58] Faron-Górecka A (2016). Chronic mild stress alters the somatostatin receptors in the rat brain. Psychopharmacology.

[59] Wolff SB (2014). Amygdala interneuron subtypes control fear learning through disinhibition. Nature.

[60] Graham B, Richardson R (2011). Memory of fearful events: the role of fibroblast growth factor-2 in fear acquisition and extinction. Neuroscience.

[61] Lu Y (2014). Maintenance of GABAergic activity by neuregulin 1-ErbB4 in amygdala for fear memory. Neuron.

[62] Alavi MS, Shamsizadeh A, Azhdari-Zarmehri H, Roohbakhsh A (2018). Orphan G protein-coupled receptors: the role in CNS disorders. Biomed. Pharmacother..

[63] Criado-Marrero M (2017). Dynamic expression of FKBP5 in the medial prefrontal cortex regulates resiliency to conditioned fear. Learn. Mem..

[64] Sargin D (2013). Disrupting Jagged1-Notch signaling impairs spatial memory formation in adult mice. Neurobiol. Learn. Mem..

[65] Sun XM, Tu WQ, Shi YW, Xue L, Zhao H (2014). Female-dependent impaired fear memory of adult rats induced by maternal separation, and screening of possible related genes in the hippocampal CA1. Behav. Brain. Res..

[66] Jacobson KA, Gao ZG (2006). Adenosine receptors as therapeutic targets. Nat. Rev. Drug. Discov..

[67] Berlacher M, Mastouri R, Philips S, Skaar TC, Kreutz RP (2017). Common genetic polymorphisms of adenosine A2A receptor do not influence response to regadenoson. Pharmacogenomics.

[68] Oertel W, Schulz JB (2016). Current and experimental treatments of Parkinson disease: a guide for neuroscientists. J. Neurochem..

[69] Shimada J (1997). Adenosine A2A antagonists with potent anti-cataleptic activity. Bioorg. Med. Chem. Lett..

[70] Shiozaki S (1999). Actions of adenosine A2A receptor antagonist KW-6002 on drug-induced catalepsy and hypokinesia caused by reserpine or MPTP. Psychopharmacology.

[71] Bleickardt, C. J., LaShomb, A. L., Merkel, C. E. & Hodgson, R. A. Adenosine A(2A) receptor antagonists do not disrupt rodent prepulse inhibition: an improved side effect profile in the treatment of Parkinson’s disease. *Parkinsons Dis.* **2012** 591094 (2012). 10.1155/2012/591094 PMC3236485 22191072

[72] Yukioka, H. et al. A potent and selective neuropeptide Y Y5 receptor antagonist, S-2367, attenuates the development of diet-induced obesity in mice. In *NAASO 2006 Annual Scientific Meeting*. Boston, Massachusetts, 2006.

[73] Shimazaki A, Tanioka H, Yokota Y, Yukioka H, Hara S, Hanasaki K. Role of Energy Expenditure in the Antiobesity Effect of Neuropeptide Y Y5 Receptor Antagonist S-2367 in Diet-Induced Obese Mice. 2007. In: The Obesity Society's 2007 Annual Scientific Meeting. New Orleans, Louisiana: Shionogi.

[74] Yulyaningsih E, Zhang L, Herzog H, Sainsbury A (2011). NPY receptors as potential targets for anti‐obesity drug development. Br. J. Pharmacol..

[75] Dawson MI, Xia Z (2012). The retinoid X receptors and their ligands. Biochim. Biophys. Acta.

[76] Missig G (2014). Parabrachial nucleus (PBn) pituitary adenylate cyclase activating polypeptide (PACAP) signaling in the amygdala: implication for the sensory and behavioral effects of pain. Neuropharmacology.

[77] McDonald AJ, Mascagni F, Guo L (1996). Projections of the medial and lateral prefrontal cortices to the amygdala: a Phaseolus vulgaris leucoagglutinin study in the rat. Neuroscience.

[78] De Bundel D (2016). Dopamine D2 receptors gate generalization of conditioned threat responses through mTORC1 signaling in the extended amygdala. Mol. Psychiatry.

[79] Aoyama S, Kase H, Borrelli E (2000). Rescue of locomotor impairment in dopamine D2 receptor-deficient mice by an adenosine A2A receptor antagonist. J. Neurosci..

[80] Farrell MS (2013). A Galphas DREADD mouse for selective modulation of cAMP production in striatopallidal neurons. Neuropsychopharmacology.

[81] Simoes AP (2016). Adenosine A2A receptors in the amygdala control synaptic plasticity and contextual fear memory. Neuropsychopharmacology.

[82] Saus E (2010). Comprehensive copy number variant (CNV) analysis of neuronal pathways genes in psychiatric disorders identifies rare variants within patients. J. Psychiatr. Res..

[83] Hohoff C (2010). Adenosine A(2A) receptor gene: evidence for association of risk variants with panic disorder and anxious personality. J. Psychiatr. Res..

[84] Paxinos, G. & Franklin, K. B. The mouse brain in stereotaxic coordinates. (Gulf professional publishing, 2004).

[85] Montojo J (2010). GeneMANIA Cytoscape plugin: fast gene function predictions on the desktop.. Bioinformatics.

