# Supplementary material for: Cell-type-specific interrogation of CeA Drd2 neurons to identify targets for pharmacological modulation of fear extinction
Source: Transl Psychiatry. 2018 Aug 22;8:164. doi: 10.1038/s41398-018-0190-y (PMC6105686; doi:10.1038/s41398-018-0190-y)
Supplement: Supplementary file 18 — Supplemental Figure Legends [file 41398_2018_190_MOESM15_ESM.docx]

**Supplemental Figure 1. Quantification of Co-Localization among *Drd2, Adora2,* and *Drd1a* across A/P axis of CeA**

Co-localization among *Drd1a, Drd2,* and *Adora2a* was quantified in the CeA male mice. Expression of *Adora2a* and *Drd2* extensively co-localized with 97.56% of *Adora2a* positive cells also expressing *Drd2* and 97.16% of *Drd2* expressing cells also expressing *Adora2a. Drd1a* expression did not co-localize extensively with *Drd2* or *Adora2a* (1.4% and .90% of cells respectively) (n=5 amygdala from different mice).

**Supplemental Figure 2. Distribution of *Drd2, Adora2,* and *Drd1a* across A/P axis of CeA**

The *Drd2/Adora2a* population is heavily represented in anterior CeC and CeL, while *Drd1a* is strongly represented in anterior CeL and CeM. Neither population is found at high levels in posterior CeA. **A-D.** Map of CeA at A/P: -.82, -1.0, -1.2, and -1.6 respectively. **E-H.** DAPI (Grey) expression at A/P: -.82, -1.0, -1.2, and -1.6. **I-L.** *Adora2a* (Green)expression at A/P: -.82, -1.0, -1.2, and -1.6. *Adora2a* is strongly expressed in anterior CeC and CeL (-8.2 to -1.2) but not in posterior CeA (-1.6). **M-P.** *Drd1a* (Red)expression at A/P: -.82, -1.0, -1.2, and -1.6. *Drd1a* is strongly expressed in anterior CeL and CeM (-8.2 to -1.2) but not in posterior CeA (-1.6). **Q-T.** *Drd2* (Cyan) expression at A/P: -.82, -1.0, -1.2, and -1.6. *Drd2* is strongly expressed in anterior CeC and CeL (-8.2 to -1.2) but not in posterior CeA (-1.6). **U-X.** Merge of channels. *Drd2* and *Adora2a* form a single co-expressing population found primarily in anterior CeC and CeL that does not co-express with *Drd1a*, which is found primarily in anterior CeL and CeM. Abbreviations used: BLA: Basolateral Amygdala, CeC: Central Capsular Amygdala, CeL: Central Lateral Amygdala, CeM: Central Medial Amygdala, Main Intercalated Island: Im. Scale Bar: A-X 200 um.

**Supplemental Figure 3. Quantification of Co-expression of *Drd2* with *Prkcd, Tac2, Sst, Crh,* and *Nts* in anterior and posterior CeA*.***

*Drd2* co-expression was quantified and represented as total number of cells expressing *Drd2*, expressing other RNA of interest, or co-expressing the two RNA’s. In all cases Mann-Whintney test was performed to determine whether the co-expressing population was significantly different from each single expressing population. * p<.05. **A.** In all regions of anterior CeA very little co-expression between *Drd2* and *Prkcd* was found. **B.** Moderate co-expression between *Drd2* and *Prkcd* was found in posterior CeC and CeL. **C-J.** Very limited co-expression was detected between *Drd2* and *Tac2, Sst, Crh,* and *Nts,* and in all cases the *Drd2* labeled population is significantly different from the co-labeled population.

**Supplemental Figure 4. Expanded y-axis view of Pre-CS freezing.**

**A.**Pre-CS freezing prior to Fear Conditioning demonstrates no difference between groups before training. This suggests no differences in baseline fear or anxiety between groups. **B.** Pre-CS freezing prior to Fear Extinction 1 demonstrates no difference between groups before fear extinction. This suggests no differences in baseline fear or anxiety between groups when CNO is on-board in the absence of explicitly fearful stimuli.

**Supplemental Figure 5. Drd2-TRAP transgene expression closely recapitulates Drd2expression pattern observed with FISH.**

**A.** L10a-GFP ribosomal subunit (Green) is expressed is pattern very similar to that observed with FISH. Abbreviations used: BLA: Basolateral Amygdala, CeC: Central Capsular Amygdala, CeL: Central Lateral Amygdala, CeM: Central Medial Amygdala, Main Intercalated Island: Im.

**Supplemental Figure 6. Fear conditioning of mice for TRAP collection.**

Mice express significantly more freezing after fear conditioning. (Unpaired t-Test, freezing to tone vs. CS1, * p < .05).

**Supplemental Figure 7. Validation of TRAP pull-down.**

**A.** Ribosomal subunit 18S is found at significantly higher levels in bound fraction, verifying ribosomal pull down (Paired t-Test, * p < .05). **B.** The ratio of *Drd2:Drd1a* is significantly higher in bound fraction vs. unbound fraction, verifying RNAs were successfully isolated from *Drd2* neurons (Paired t-Test, * p < .05).

**Supplemental Figure 8. Gene set enrichment analysis.**

Using the entire expression dataset in default setting genes identified in *Drd2-*TRAP fear conditioning study and humanized model of 22q11.2 deletion are significantly and concordantly regulated in inverse direction in both **A.** Prefrontal cortex and **B.** Hippocampus.

**Supplemental Figure 9. Replication of pharmacological manipulation of fear extinction with Istradefylline.**

**A.** Schematic of experimental design. **B.** Two groups of animals were fear conditioned (5 CS/US, .65 mA). **C.** Animals were injected with Vehicle or Istradefylline 30-minutes before fear extinction. Animals injected with Istradefylline froze significantly less than those injected with vehicle (2-way RM ANOVA, F(1,18)=27.94, p < .0001). **D.** Animals previously injected with Istradefylline froze statistically less than Vehicle control during second fear extinction session 24-hours later(2-way RM ANOVA, F(1,18)=5.8, p = .028).

**Supplemental Figure 10. Replication of pharmacological manipulation of fear extinction with Velneperit.**

**A.** Schematic of experimental design. **B.** Two groups of animals were fear conditioned (5 CS/US, .65 mA). **C.** Animals were injected with Vehicle or Velneperit 90-minutes before fear extinction. Animals injected with Velneperit froze significantly more than those injected with vehicle (2-way RM ANOVA, F(1,13)=13.85, p =.0026). **D.** Animals previously injected with Istradefylline showed no statistical difference in freezing compared to Vehicle control during second fear extinction session 24-hours later (2-way RM ANOVA, F(1,13)=2.262, p = .15).

**Supplemental Figure 11. Examination of *Drd2* expression following fear conditioning and extinction.**

**A.** Two groups of animals (FC1 and FC 30) were fear conditioned (5 CS/US, .65 mA US). **B.** Twenty-four hours three groups of animals were exposed to extinction context. FC1 was exposed to a single CS in extinction context. Percent freezing indicates freezing during tone during CS 1 and freezing during the corresponding time when tone was played for other groups for CS 2-30, freezing during CS 1 was significantly greater than control (unpaired t-Test F(1,12)=5.929, p < .0001). HC 30 group did not receive fear conditioning, but was exposed to 30 CS’s in extinction context. FC 30 was exposed to 30 CS’s in extinction context and expressed more freezing than HC 30 group (Each bin examined by students t-test p < .05). **C.** Four groups were sacrificed 2 hours following behavior and qPCR performed on amygdala punches. FC30 group had significantly increased *Drd2*expression compared to all other groups.

**Supplemental Table 1. Complete list of differentially regulated RNA’s.**

LogFC indicates log fold change between FC and Control group. Negative LogFC’s indicate decreased expression compared to control while positive values indicate increased expression. FDR indicates false discovery rate corrected across multiple tests. Only q< .05 values are featured.

**Supplemental Table 2.**

Using Mouse Gene Atlas dataset, Enrichr confirms amygdala specificity of pull-down and gene change.

**Supplemental Table 3. Jensen COMPARTMENTS analysis.**

Jensen COMPARTMENTS analysis data set, using sequence based prediction methods confirms neuronal specificity of pull-down and gene change.

**Supplemental Table 4. Drug-Gene analysis of differentially expressed genes.**

Metacore ‘Drug for Drug database’ identifies drugs that target protein products of differentially expressed genes.
